# Expression of the Epigenetic factor BORIS (CTCFL) in the Human Genome

**DOI:** 10.1186/1479-5876-9-213

**Published:** 2011-12-14

**Authors:** Rosalia de Necochea-Campion, Anahit Ghochikyan, Steven F Josephs, Shelly Zacharias, Erik Woods, Feridoun Karimi-Busheri, Doru T Alexandrescu, Chien-Shing Chen, Michael G Agadjanyan, Ewa Carrier

**Affiliations:** 1 Division of Hematology and Oncology, School of Medicine, Loma Linda University, Loma Linda, CA 92354, USA; 2 Department of Medicine, University of California, San Diego, CA 92093, USA; 3 Department of Molecular Immunology, Institute for Molecular Medicine, Huntington Beach, CA 92647, USA; 4 Therinject LLC, San Diego, CA 92121, USA; 5 RenovoCyte LLC, Indianapolis, IN 46202, USA; 6 NovaRX Corporation, Stem Cell Dept., San Diego, CA 92121, USA; 7 Department of Dermatology, Georgetown Dermatology, Washington, DC 20017, USA; 8 University of California, Irvine, Institute for Memory Impairments and Neurological Disorders, Irvine, CA 92697, USA; 9 Mechnikov Research Institute of Vaccine and Sera, Russian Academy of Medical Sciences, Moscow, 105064, Russia

**Keywords:** BORIS, CTCF, epigenetic regulation, protein partners, cancer immunotherapy

## Abstract

BORIS, or CTCFL, the so called Brother of the Regulator of Imprinted Sites because of the extensive homology in the central DNA binding region of the protein to the related regulator, CTCF, is expressed in early gametogenesis and in multiple cancers but not in differentiated somatic cells. Thus it is a member of the cancer testes antigen group (CTAs). Since BORIS and CTCF target common DNA binding sites, these proteins function on two levels, the first level is their regulation via the methylation context of the DNA target site and the second level is their distinct and different epigenetic associations due to differences in the non-homologous termini of the proteins. The regulation on both of these levels is extensive and complex and the sphere of influence of each of these proteins is associated with vastly different cellular signaling processes. On the level of gene expression, BORIS has three known promoters and multiple spliced mRNAs which adds another level of complexity to this intriguing regulator. BORIS expression is observed in the majority of cancer tissues and cell lines analyzed up to today. The expression profile and essential role of BORIS in cancer make this molecule very attractive target for cancer immunotherapy. This review summarizes what is known about BORIS regarding its expression, structure, and function and then presents some theoretical considerations with respect to its genome wide influence and its potential for use as a vaccine for cancer immunotherapy.

## Introduction

BORIS is a complex and highly versatile transcription factor sporadically expressed in numerous mammalian cells and member of the cancer-testis antigen (CTA) family, a group of genes expressed in the testis and abnormally expressed in cancer malignancies [1]. Various studies have attempted to elucidate the role of BORIS in different cell types, however the exact mechanisms by which it interacts with the genome and the extent to which it influences cellular processes remain largely a mystery. BORIS appeared fairly early in the evolutionary tree most likely through a full-length genomic duplication of CTCF before the mammalian-reptilian split [2] which occurred about 310 million years ago [3,4]. BORIS is the only known paralog of CTCF a protein that has been called the "master weaver of the genome"[5], and for which a staggering 14,000--25,000 potential binding sites have been identified in the human genome [5-7]. Both of the proteins have a central 11 zinc finger the DNA binding region with very similar amino acid sequence which has conserved more than a 74% residue identity in humans [2,8]. This indicates a tightly controlled evolutionary selection process to conserve a highly specific genome-wide DNA binding ability which suggests a very important and likely critical role for BORIS in chromatin functions. Indeed, BORIS has been implicated in numerous regulatory functions including cell proliferation [9], activation of other CTA genes [10,11], spermatogenesis [12], and human preimplantation development [13], however the extent and the importance of it's cellular role is still not well understood. The difficulty in defining this role is due in part to the fact that BORIS expression is inconsistent in many cells, particularly cancer (Table 1). An evaluation of the functional characteristics of the BORIS promoter region has shown that BORIS expression can be repressed by at least three things: DNA methylation, the presence of CTCF or the presence of p53 [14]. Furthermore, the BORIS promoter region actually contains three differentially regulated promoters which control expression of the BORIS gene and produce 5 alternatively spliced mRNAs [14]. Initially, those authors hypothesized that the function of the 5' mRNA variants was related to transcript stability since they all seemed to generate the same protein product despite differences in their 5' non-coding regions. More recently the same research group identified a much larger number of alternatively spliced BORIS mRNA variants detecting a total of 23 different BORIS isoforms expressed in human cells [8]. Some of these isoforms encode specific amino and carboxyl translational frame shifts or produce proteins with distinct zinc-finger combinations. Undoubtedly, all of these characteristics can affect the specific chromatin regulatory functions of BORIS. This seems to be supported by the finding that distinct isoform expression patterns are detected in different cell types [8].

**Table 1:** Variability in the frequency of BORIS expression detected in cancer (NT = not tested)

Type of Cancer	Cell Line Expression	Primary Tumor Sample	References
Breast	8%-100%	71%*-92%*	[11]* [14][49]* [42]

Melanoma	90%-100%	27%*	[11][14][43]*

Colon	75-100%	80%*	[11]* [14]

Prostate	50%-60%	90%*	[11]* [62]

Ovarian	40%-100%	NT	[14,39]

Lung	60%-100%	NT	[11][14]

Leukemia	100%	NT	[14]

Bladder	27%	NT	[62]

Uterine	77%	NT	[6]

Endometrial	77%	NT	[6]

Osteosarcomas	NT	38%*	[63]*

Squamous cell carcinomas	NT	81%*	[54]*

### DNA Binding Ability

Most of what is known about the specific way that BORIS utilizes combinations of zinc fingers (ZFs) to bind to DNA has been inferred from studies done with CTCF which found that specific combinations of zinc-fingers are used to bind to highly diverse target sequences. This was determined through extensive experimental analyses involving sequential deletion of ZFs and subsequent characterization of mutant CTCF nucleotide interactions [15]. Due to the high degree of sequence similarity, we assume that BORIS uses the same ZF combinations when binding the same specific target sequences, however other factors may influence this protein's overall ability to bind to certain genomic regions. The recently characterized isoforms of the BORIS transcript were found to include one splicing variant which codes for a protein containing an entirely new zinc finger formed by splicing together two ZF halves (ZF4 and ZF9)[8]. The 23 BORIS isoforms were found to translate into 17 distinct polypeptide products and the ability of these 17 proteins to bind to 2 specific genomic regions was studied. These authors found that 9/17 isoforms could bind to a specific IGF2/H19 imprinting control region (ICR) target probe, while 13/17 isoforms could bind to a testis-specific CST gene promoter. This difference made it possible to determine that 9 of the BORIS zinc-fingers were involved in binding to the H19 ICR site while only 5 were needed for binding to the CST promoter.

### DNA binding properties of BORIS compared to CTCF

Several genomic factors may be involved in determining whether BORIS or CTCF binds to a specific region, however numerous studies suggest that DNA methylation plays an important role in this process. Most studies find that CTCF does not bind to sequences of DNA when they are methylated, such as regulatory regions of hTERT [14] and rDNA [16], or insulator regions surrounding MHC-II genes [17]. A few authors have compared the binding properties of both BORIS and CTCF. In one study that analyzed the SCA 7 locus, it was reported that neither BORIS nor CTCF could bind to methylated DNA sequences [18]. Another study which analyzed histone methylation patterns in the BAG-1 promoter region, found CTCF binding to be associated with a nonpermissive chromatin status characterized by low dimethyl-H3-K4/dimethyl-H3-K9 ratio while BORIS binding appeared to coincide with changes that resulted in a permissive chromatin status [19]. Intriguingly, these authors did not find any significant differences in the level of DNA methylation in the BAG-1 region associated with BORIS or CTCF binding.

There has been some controversy in the literature regarding the extent of the influence of DNA methylation over BORIS and CTCF binding ability, however only a few target sites have really been analyzed in detail. One of these is the IFG2/H19 ICR where it was found that neither CTCF nor any of the 9 BORIS isoforms that could bind to this target site, could do so after the H19 ICR probe was methylated in vitro by SssI methyltransferase [8]. In contrast, others reported that BORIS binding to this region was largely methylation insensitive since a large number of BORIS-DNA interactions were observed in methylated DNA segments from this region [20]. This discrepancy may arise from the use of different experimental and analytical techniques. Whereas one group used EMSA assays with labeled probes specific to the 6^th ^CTCF target site in the H19-IGF2 [8], the other group performed ChIP assays that compared BORIS and CTCF binding to genomic DNA fragments containing a 360bp segment from the region of interest [20]. With ChIP assays it is difficult to determine the exact binding location within the area that is being analyzed [21], so it is possible that the BORIS binding observed in this experiment was in a non-methylated segment. However, these authors [20] then carried out in vitro experiments where they did observe BORIS binding to an SssI-methylated H19 CTCF binding site probe. So it is possible that the influence of DNA methylation over BORIS vs. CTCF binding is very site specific. This is particularly evident in another study where the authors analyzed DNA methylation patterns of 23CpG dinucleotides over a 700bp segment of the MAGE-A1 regulatory region and determined their influence on CTCF and BORIS binding to this region [11]. Surprisingly, they found that strong CTCF binding occurred in vivo when the area was largely methylated, and that binding could also occur to a methylated target probe from this region in vitro. Furthermore, the switch from CTCF to BORIS occupancy occurred when the area was demethylated either by 5azadC treatment or conditional expression of BORIS through transient transfection [11]. Thus the presence of BORIS was associated not only with CTCF displacement but also with further demethylation of the MAGE-A1 regulatory region and activation of gene expression [11]. In summary, many questions remain about the specific mechanisms connected with BORIS and CTCF binding abilities and to understand why the influence of DNA methylation is so prevalent under some circumstances and not others. Much investigation is still needed to understand how DNA methylation of specific sequences or regions relative to the specific CTCF/BORIS binding site can influence binding ability as well as to understand how this may vary among distinct BORIS isoforms.

### Gene Regulation and Protein Partners

**Figure 1:**
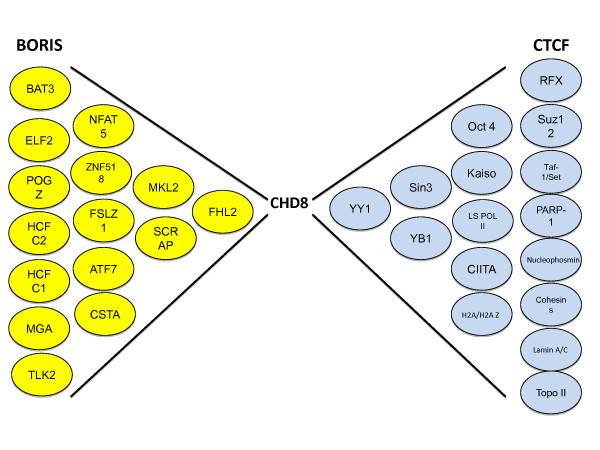
**Known human interacting protein partners of BORIS (yellow) and CTCF (blue). Squares indicate the proteins observed to interact with both BORIS and CTCF**. van de Nobelen *et al*., 2010 identified UBF as a common interacting partner for both BORIS and CTCF [16]. All other proteins shown to associate with BORIS were identified by Nguyen *et al*., 2008 and isolated using yeast 2-hybrid screening techniques with N-terminus segments of the BORIS protein as bait [25]. Proteins shown to interact with CTCF were all discussed in an excellent review published by Ohlsson *et al*., 2010 [27].

Some studies have shown that the effects of BORIS binding to specific genomic regions are very different from those of CTCF. In fact, these proteins generally exert exact opposite effects over gene expression. Typically BORIS is found to activate gene expression and be involved in cell proliferation, while CTCF represses gene expression and inhibits cell proliferation [9,20]. Specifically, BORIS has been reported to activate and CTCF to represses expression of MAGE-A [11], BORIS [14], BAG-1 [19], NY-ESO-1 [22], and hTERT [23]. It is not surprising that these proteins are associated with opposing functional outcomes, given the significant sequence and structural differences of their amino and carboxyl extremes. A comparison of BORIS and CTCF reveals that in spite of the high degree of similarity (74.1% identity) conserved in the DNA binding region, the ends of the proteins share less than 15% sequence identity [2]. Furthermore, both CTCF and BORIS are known to associate with an extensive list of different protein interacting partners (Figure 1) which essentially change the structure of the DNA-bound protein complex, and presumably modify its specific activity and chromatin regulating abilities. Therefore, it may actually be the protein partners of CTCF and BORIS that ultimately determine their specific cellular function at a specific location. The importance of these interactions seems to be supported by the fact that an analysis of the amino and carboxyl protein domains of BORIS shows them to be largely unstructured molecules suggesting that their architecture may be organized through their protein associations [24].

In Figure 1 we see many of the human interacting protein partners of BORIS which were identified using yeast 2-hybrid isolation techniques [25]. Several BORIS N-terminus segments of varying length were used as bait and most of the isolated proteins were able to bind many of these bait targets. This makes it likely that several of the BORIS protein partners interact with more than one of its 17 paraprotein alternative splicing variants. It was reported that almost all of these BORIS splicing variants conserve a 258 base pair N-terminus region [26], which may demonstrate the importance of conserving the protein interactions that occur within this protein domain. It is interesting to note that when the specific interacting partners of the N-terminus of BORIS are compared with interacting partners of CTCF, only CHD8 is identified as an interacting partner of both (Figure 1). Considering that CHD8 is found to interact with both the unique N-terminus sequence of BORIS [25], and the central zinc finger region of CTCF [27], it is intriguing to speculate on the distinct genomic role that may result from these distinct interactions. It is thought that the role of the CTCF-CHD8 interaction regulates the insulator function of CTCF [28], but the effects of it's interaction with BORIS have not been studied. It is possible that some of the interacting partners known to associate directly with the zinc finger DNA binding domain of CTCF, such as Sin3A [29,30], YB-1 [31], and Oct4 [32] may also interact with the same region of the BORIS protein due to it's sequence similarity. In fact, a common zinc finger region interacting partner, UBF, was recently identified [16] (Figure 1). UBF forms part of the RNA polymerase I Complex which transcribes ribosomal RNA [16]. It is believed that the CTCF-UBF interaction may help prepare ribosomal DNA for transcription [16], but the effects of the BORIS-UBF interaction are yet to be studied.

### Epigenetic Regulatory Mechanisms

Although the importance of the BORIS interacting protein partners has been recognized, very few studies have actually attempted to elucidate the specific function of these protein associations. One study found that BORIS acted as a scaffold onto which the BAT3 and then SET1A proteins were recruited to the *myc *and BRCA1 promoters [19]. It was shown that this protein complex increased H3K4 methylation and promoted gene expression at these two pro-carcinogenic promoters. A second study analyzed BORIS interactions in the mouse genome and found that it recruited the PRMT7 protein to the H19 ICR site in the developing testis [33]. PRMT7 is a protein arginine methyltransferase known to catalyze specific chromatin histone methylations [33,34]. It was shown that the BORIS could bind to both the H2 and H4 histones at the H19 ICR site and the BORIS-PRMT7 interaction directly correlated to an enrichment in symmetrical dimethylation of arginine 3 at histone 4(H4R3me2)[33]. A third study found both BORIS and Sp-1 proteins present at NY-ESO-1 promoters in lung cancer cells [10]. After demonstrating that these proteins directly interacted, these authors concluded that BORIS recruits Sp-1 and forms a transcriptional regulatory complex that activates NY-ESO-1 expression in these cancer cells. In each of these studies it was concluded that the presence of BORIS was essential for proper positioning of the other proteins. This leaves us to question if the primary regulatory role of BORIS could be to function as a type of chromatin beacon which attracts and orients other proteins properly in the genome.

**Figure 2:**
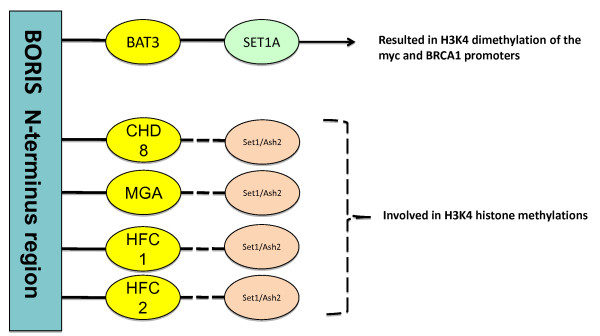
**Shown are the proteins known to bind to the N-terminus region of the BORIS which have the potential to interact with methyltransferases with catalyze H3K4 methylations**. Solid lines represent documented protein complexes [25], and dashed lines represent the potential for additionally formed protein complexes since it is unknown if these interactions [35] also take place in the presence of BORIS.

A primary role for BORIS as a genomic recruiter seems likely given the number of nuclear proteins known to interact with it's unique N-terminus sequence (Figure 1), the fact that a 258 residue N-terminus segment highly conserved among all of its recently discovered isoforms [8], and the difficulty in understanding and defining the specific cellular role of BORIS despite its discovery about 10 years ago [26]. We propose that in addition to some of the specific regulatory functions that have been reported for BORIS, it also modulates epigenetic changes on a massive scale. This is due to the fact that many of the protein partners that interact with BORIS are also known to interact with histone methylating complexes (Figure 2). It is possible to envision a scenario in which specific isoforms of BORIS bind to DNA, and then attract specific interacting partners, which in turn attract and ultimately organize methytransferase complexes correctly in the genome. This could result in epigenetic remodeling on a massive or even a very specific scale depending on the abundance or role of a particular BORIS isoform as well as the affinity for a specific interacting partner, and the likelihood that this partner then associates with methyltransferases as depicted in Figure 2. It is known that the specific methyltransferase complex known to associate with the potential interacting partners of BORIS [35] does catalyze H3K4 dimethylations associated with active gene transcription [36-38]. And the presence of BORIS in specific genomic locations is generally linked to active gene expression. In addition, some studies have analyzed the correlation between BORIS expression and H3K4 chromatin methylation status demonstrating a connection to specific epigenetic changes. It was found that cellular conditions that increase expression of BORIS are linked to increased levels of H3K4me2 in the BORIS promoter region [39], known to contain several BORIS binding sites [14]. Suppression of BORIS expression has an opposite effect. Knockdown of BORIS expression in human colon carcinoma cell lines decreases H3K4 methylation as well as expression of myc, BRCA1 and H19 [25] Another study found that suppression of BORIS expression decreases H3K4 methylation at the BAG-1 promoter resulting in decreased expression of this gene [19]. In all, the results of these studies suggest that BORIS may play a central role in genome wide histone methylation modifications.

### Role in the Testis

Part of the difficulty in understanding the cellular role of BORIS has been the increasing number of diverse cell types in which BORIS expression has been documented. Traditionally, "normal" expression of BORIS was described as being confined to the testis and involving a role in spermatogenesis [9,14]. It was thought that since this is the only place where CTCF is not expressed [9], DNA-binding competition could be avoided between these proteins, and thus all BORIS activity could be linked to normal functions. Still, the role of BORIS in spermatogenesis has not been well defined. It has been speculated that the role of BORIS in male germline cells involves the epigenetic regulation responsible for DNA methylation of imprinting control regions [33]. Also it has been seen to bind to and activate the promoter of a cancer-testis gene, SPAN-X [6]. Recently it was shown that BORIS knockout mice have smaller testes and defective sperm production, although surprisingly they are still fertile [12]. The absence of BORIS causes a dramatic reduction in the expression of the CST gene, which has a critical role in meiosis and causes a significant delay in sperm production [12]. In all, these studies indicate that in testis tissues BORIS regulates gene expression and may play an important role in meiosis necessary to produce the haploid sperm.

### Role in Cancer

Expression of BORIS in cancer has traditionally been viewed as an aberrant phenomenon although there has been some controversy regarding the importance of its role in these cells. In epithelial ovarian cancer the *BORIS/CTCF *expression ratio is also associated with increased stage and decreased overall and progression-free survival [40]. It has been suggested that the expression of BORIS displaces CTCF in the genome and leads to proliferation of cancer cells [26]. The results of some studies seem to support this theory because BORIS has been seen to bind to the promoters and activate expression of several CTA oncogenes [11,22]. Intriguingly, one study reported that silencing BORIS expression in a breast cancer cell line has a concentration dependent apoptotic effect on these cells [41]. In contrast, another publication reports that BORIS is not expressed in the majority of breast cancer cell lines or tumors and is unlikely to play a role in tumorigenesis [42]. These authors propose that the use of sensitive quantitative techniques to measure BORIS expression may be a reason for their contradictory findings. However, others have found that even when BORIS expression is forced in cancer cells it does not automatically lead to the activation of the cancer-testis genes it is proposed to regulate [43,44]. One possibility is that the presence of BORIS alone is insufficient to displace CTCF binding and repression over CTA gene expression in the genome. Another possibility is that a particular isoform of BORIS is necessary for the regulation of these CTA genes. In fact, it is only when a general expression of BORIS is induced in cancer cells by the addition of the DNA-demethylating agent 5-azadC [10,11,22], as opposed to using genetic constructs [43,44] that it is associated with the activation of CTA genes. These conflicting findings are difficult to interpret due to a lack of knowledge about the roles of different BORIS isoforms, their potential interacting partners, and the genes they regulate or influence. Interestingly, the entire genomic region of the BORIS locus was studied recently and two minisatellite loci (BORIS-MS1 and BORIS-MS2) upstream of BORIS have been identified [45]. In addition, analyses of allelic variations in the minisatellites of BORIS were found to be significantly related to susceptibility to breast cancer in young patients within the Korean population studied [45].

Although the detection of BORIS in cancers seem to be well documented, other important questions also remain to be answered, including how frequently BORIS is expressed in different cancers and how much BORIS is expressed per cell. Some authors argue that the expression of mRNA below 1 copy per cell should be ignored [43]. This statement may be true if all cancer cells were to express BORIS. However, it is a common knowledge that cancer cells within a tumor are very heterogeneous and only a fraction of cells may express BORIS then the question would be what is this fraction of BORIS expressing cells and how important is it for cancer development. Other authors suggest that in order to compete with CTCF at certain sites BORIS should be expressed at levels comparable with CTCF [42]. However, it is extremely unlikely that the amount of BORIS be at the same level as CTCF as it will compete out CTCF at majority of its binding sites creating a situation similar to CTCF knock-out that is reported to be lethal to cells. Theoretically, if only few CTCF sites are occupied by BORIS that might be sufficient to achieve growth advantage over the cells that maintain CTCF binding on these sites. But that means that very low expression by BORIS might be enough to compete out CTCF from few sites provided nuclear compartmentalization selectively delivers BORIS protein to some sites and not to the other. Moreover, there are two publications that reported selective loss of CTCF binding from p16^ink4a ^locus [46] and p53 promoter [47]. Clearly, genome-wide ChIPs with good anti-BORIS antibodies on tumor cell lines and primary tumors will answer those important questions.

### BORIS expression in other cell types

Analysis of BORIS expression in human cells and tissues has revealed several other cell types that express this protein. These include the ovary, leukocytes from breast cancer patients, and embryonic stem cells. Similar to it's function in spermatogenesis, BORIS expression in the ovary is thought to have a role in meiosis during oogenesis [13]. The expression of BORIS in oocytes and it's disappearance in cleavage stage embryos is found to coincide closely with genome-wide events of DNA demethylation (oocytes) and then re-methylation (cleavage stage embryos)[48]. BORIS expression can also detected in leukocytes isolated from the blood of breast cancer patients [49]. Here quantities of BORIS protein correlate strongly with tumor size and thus it's potential for use as a cancer biomarker has been evaluated [49]. It is possible that BORIS may play a role in the antitumoral response of these cells however its exact function is unknown. Again, part of the difficulty in understanding the role of BORIS in these diverse cell types can be attributed to a lack of knowledge about the expression patterns of specific isoforms and their extensive protein interactions which probably define the specific cellular function of BORIS at a certain moment or specific genomic location.

BORIS expression is not detected in cleavage stage embryos or human blastocysts, however it can be detected in human embryonic stem (hES) cells isolated from their inner cell mass [13]. Moreover, it was shown that BORIS expression disappears upon hES cells differentiation [8]. The function of BORIS in human embryonic stem cells is not clear, however, it is thought to be associated with epigenetic programming events involving pluripotency [13]. Intriguingly, BORIS protein is seen to co-localize with self-renewal proteins NANOG and OCT4 in the nucleus of these cells [13]. However, adaptation of hES cells to prolonged growth in culture is associated with gradual loss of multilineage differentiation capacity along with gradual gain of chromosome 20 (BORIS is mapped at 20q13), increased levels of BORIS expression and MAGEA4 activation [50]. Consistent with this is that the levels of BORIS mRNA were increased more than 10-fold (observed in both passage 132 and 237) upon long term maintenance of H7 hES cells in culture [50]. Throughout this process of hES "adaptation to growth in culture" a profound loss of pluripotency was observed concomitant with loss of the q arm of chromosome 16 (the locus of CTCF) and gain of chromosome 20q13 the genetic location BORIS. This 20q13 gain as well as 16q22 loss of heterozygosity (LOH) with increased BORIS expression parallels a similar association in cancers [51] that also show CTCF haplo-insufficiency [52,53]. It is possible that a 16q LOH may result in a reduction of CTCF which is known to repress BORIS expression, as does p53 and DNA-methylation [14]. In this situation these suppressive effects might also decrease since BORIS expression is commonly associated with events of global demethylation [22,54]. Under these circumstances it is possible that even a slight elevation of BORIS may exert greater overall epigenetic influence than in a normal situation where increased competition with CTCF may prevent DNA binding. Furthermore, this may help explain the significant fluctuations in the levels of BORIS that coincide with many primary cancers (Table 1). Further analyses will be required to address this interesting cancer-associated phenomenon.

### BORIS as a vaccine for cancer immunotherapy

**Figure 3:**
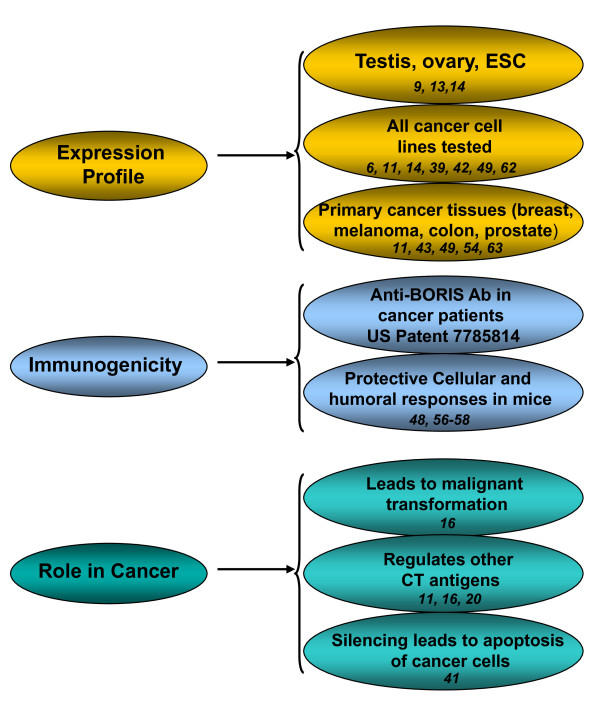
**BORIS characteristics that make it an attractive target antigen for cancer treatment**. Numbers indicate numeric references associated with each category.

Recent studies have provided proof of concept for the utility of anti-cancer immunotherapy strategies in several clinical settings, although with modest efficacy. The major issues of cancer immunotherapy are the tendency of tumor cells to escape immune surveillance by inducing tolerance to self-antigens, changing antigenic profiles and producing immune suppressing agents. Therefore, availability of strong and immunogenic tumor associated antigens and combination of immunotherapy with the agents inhibiting immune suppression could improve the efficacy of immunotherapy significantly. The ideal characteristics for candidate antigens for cancer immunotherapy are the tissue distribution that is restricted to tumors, the immunogenicity, and the essential contribution of the antigen in the transformation and maintenance of the malignant phenotype of tumor cells. Characteristics of BORIS molecule described above and in Figure 3 provide evidence that BORIS is an attractive target antigen for immunotherapeutic strategies to treat different types of cancer. Data presented in Table 1 demonstrate that BORIS expressed in a high percent of primary tumors of different origin including breast, melanoma, colon, and prostate. As a cancer testes antigen, it has a strong rational particularly in tumor types affecting predominantly females due to the minimal state of intrinsic tolerance. Anti-BORIS antibodies were detected in the sera of breast cancer patients (United States Patent 7785814), confirming its immunogenicity and proving it as a valid target for anti-tumor immunotherapy.

The characteristics of BORIS molecule described above along with the preclinical work performed up today resulted in BORIS being included in the list of high priority TAAs generated by the NCI Translational Working Group [55]. However, development of BORIS-based anti-cancer vaccine requires certain safety concerns to be met. Specifically, because of the DNA binding and gene activating functions as well as epigenetic reprogramming function of BORIS, administration of the functional BORIS protein would pose a hypothetical risk of BORIS accelerating the progression of cancer. To alleviate such safety concerns, a target antigen based on BORIS molecule that lacks the DNA-binding ZF domain have been generated. To evaluate the potency and magnitude of the immune response, class of immune response, and to select a particular strategy for eliciting anti-tumor immunity we generated and tested DNA (pmBORIS), protein (mBORIS) and adenoviral (AdBORIS) vaccines based on truncated mouse BORIS [56-58]. As was expected, protein-based vaccine induced high titer of BORIS-specific antibody and Th2 type of cytokine responses, but not cytolytic activity. In contrast, DNA and adenovirus based vaccines induced potent antigen-specific Th1-type response and cytolytic responses. Molecular adjuvants, plasmids expressing the cytokines IL-12 and IL-18, augmented the antigen-specific cellular immune responses in mice immunized with pmBORIS. Importantly, generated immune responses mediate cytotoxicity to a wide range of histologically unrelated tumors (including breast cancer, glioma, plasmacytoma) in an MHC class I-dependent manner [41]. The protective potency of pmBORIS vaccine was evaluated in a 4T1 stringent mouse model of spontaneous mammary carcinoma that closely resembles human breast cancer. Vaccination with pmBORIS along with pIL12 and pIL18 immunomodulators as well as with AdBORIS prior to tumor challenge inhibited mouse mammary tumor growth and prolonged survival of vaccinated mice [56][58]. Although prophylactic vaccination is important for initial proof of concept and efficacy studies, therapeutic vaccination more accurately reflects the clinical situation. Our data demonstrate that therapeutic vaccination of mice with mBORIS-based vaccine after implantation of poorly immunogenic and very aggressive 4T1 adenocarcinoma significantly inhibits tumor growth and progression of metastatic disease compared with control mice [59]. Importantly, infiltrations of both CD4^+ ^and CD8^+ ^T cells were significantly increased, while the numbers of myeloid derived suppressor cells (MDSC) were significantly decreased in tumors, but not in spleens of vaccinated mice compared to the control ones. Even in the absence of changes in the splenic MDSC and T_REG _populations, vaccination led to the significant inhibition of tumor growth and metastatic disease arguing for strategies that not only elicit strong anti-cancer immunity, but also robust inhibition of suppressor environment. Taken together these various observations suggest that BORIS is immunogenic, likely expressed early in and contributes to the transformation and in the maintenance of the malignant phenotype, has a wide expression profile in multiple tumor types whereas the expression is restricted to testes in normal tissues. All these features make BORIS very promising as a target antigen suggesting that BORIS-based therapeutic vaccine strategies combined with agents attenuating tumor associated immune suppression will be very effective in clinical settings. Another strategy is to use BORIS based vaccine for prophylactic/preventive vaccination of healthy patients with genetic susceptibility to cancer. For example using breast cancer diagnostic biomarkers such as BRCA1 [60], BRCA2 [61], and *BORIS*-MS2 [45] one could identify the high risk population and potentially treat with preventative vaccination. Ultimately, antigen based immunotherapy approaches involving ideal target candidates such as BORIS guided by enhanced genetic analyses may be the future of cancer medicine and useful for the treatment of cancer of multiple origins.

## Conclusion

In this review we describe a unique molecule, BORIS, which has a highly conserved 11 zinc finger DNA-binding domain very similar to its better known paralog, CTCF. Although thousand papers are published after the discovery of CTCF very little is known about BORIS. These two proteins target common DNA binding sites but BORIS is only expressed in early gametogenesis and in multiple cancers, not in differentiated somatic cells. Evidence indicates that BORIS has an extensive regulatory role in the cell through complex epigenetic associations that are influenced by DNA methylation as well as numerous protein interactions. The expression profile and regulatory role of BORIS in cancer makes this an attractive antigen target in cancer immunotherapy. Several promising approaches toward the development of a BORIS based cancer vaccines are being investigated and are likely to be effective in the treatment of diverse cancers.

## Authors' contributions

RN wrote and prepared the manuscript, AG and SJ wrote a significant portion of the manuscript and helped prepare the figures, SZ, EW, FK, DA and CSC participated with significant intellectual and written contributions that helped shape and modify the content of the manuscript, MA carried out critical revision and re-writing, EC made significant contributions to all aspects of manuscript preparation and approved the final draft. All authors read and approved the final manuscript.

## Competing interests

The authors declare that they have no competing interests.
